# Improving On-Call Support for Doctors: A Quality Improvement Project

**DOI:** 10.1192/bjo.2024.350

**Published:** 2024-08-01

**Authors:** Sharna Bennett, Verity Williams, James Anslow, Mohan Bhat

**Affiliations:** Kent and Medway NHS and Social Care Partnership Trust, Kent, United Kingdom

## Abstract

**Aims:**

Doctors completing on-call shifts at sites across a mental health trust identified a need to improve aspects of on-call work. This quality improvement project (QIP) aimed to improve response to trainee concerns arising from on-call work and support to junior doctors on-call.

**Methods:**

A previous QIP cycle identified trainee concerns regarding on-call processes. In our first QIP cycle, surveys were sent to all consultants and SpRs working on non-residential on-call rotas, and Foundation, GP and Core Psychiatry trainees (on residential on-call rotas) in the Trust, regarding perceptions of on-call processes, senior support and on-call issues. A monthly, online forum was introduced in August 2023 to improve on-call feedback and communication. Trainees, consultants and SpRs from 2 localities were invited, along with representatives from the medical staffing team, medical education team and medical management. After 4 forums, participants who had attended an on-call forum were sent a further feedback survey collecting quantitative and qualitative data. Subsequently, forum frequency and scheduling were amended, advertisement improved, and the forum was expanded to include on-call doctors across the whole Trust.

**Results:**

First cycle data revealed consultant support for a regular meeting with trainees and senior colleagues to bring issues from on-calls for discussion (56% felt that an on-call forum would be helpful, 33% felt it might be helpful). Mean forum attendance was 14, with attendance from all grades. Feedback data from trainees (5 responses) was that most found the forum useful (80%); 80% felt listened to; all felt able to raise concerns, and all wanted the forums to continue. Qualitative feedback included: ‘*we started a new QI project from the forum and many on-call guidelines became more defined.*’ Consultant feedback (4 responses) was that most found the forums useful (75%); 100% gained a better understanding of trainee concerns; 100% thought forums should continue, although 50% thought the frequency should be reduced. Most consultants and trainees did not feel it would be useful to discuss clinical cases in the forums. Consultant qualitative feedback reported that the forum was helpful to understand trainee concerns, but there should be wider attendance.

**Conclusion:**

Establishing an on-call forum was a valuable intervention for both consultants and trainees working on an on-call rota and has led to a further quality improvement project. Respondents felt that clinical supervision offered sufficient space to discuss clinical cases. Increasing trainee and consultant engagement with the forum is the next phase of this project.

